# The Melon Sterol Transporter Niemann-Pick C1 Protein Is a New Interactor of *Cucumber mosaic virus* Movement Protein

**DOI:** 10.3390/v18050577

**Published:** 2026-05-20

**Authors:** Núria Real, Irene Villar, Bin Liu, Manale Gajjout, Weina Hou, Ana Montserrat Martín-Hernández

**Affiliations:** 1 Centre for Research in Agricultural Genomics (CRAG) CSIC-IRTA-UAB-UB, C/Vall Moronta, Edifici CRAG, Bellaterra (Cerdanyola del Vallés), 08193 Barcelona, Spain; nuria.real-tortosa@jic.ac.uk (N.R.); ivillarua@gmail.com (I.V.); liubincau@163.com (B.L.); manale.gajjout.97@gmail.com (M.G.); hwn8980@gmail.com (W.H.); 2 Institut de Recerca i Tecnologia Agroalimentàries (IRTA), Campus UAB, Bellaterra, 08193 Barcelona, Spain

**Keywords:** Niemann-Pick C1, *Cucumber mosaic virus*, movement protein, interactor domain, virus infection, melon

## Abstract

Plant viruses need to use many host factors to establish infection. During the viral cycle, intracellular transport is fundamental to reach the plasmodesmata to enable cell-to-cell transport. *Cucumovirus CMV* (cucumber mosaic virus, CMV) can infect plants from most economically important crops. To identify additional host proteins involved in CMV movement in melon, we used the MP as a bait to screen a Yeast two-hybrid cDNA library from CMV-infected plants and identified a Niemann-Pick C1 (NPC1) protein as a novel MP interactor. NPC1 is a transmembrane protein involved in cholesterol transport in animal cells, but also in the infection by several viruses of different families. The identified clone from the melon *NPC1* gene spans from exons 25 to 28 and includes two introns. Notably, deletion of the two introns and exon 28 does not impair the interaction capacity of the remaining peptide. The identified *CmNPC1* gene maps to chromosome 11. In addition, the melon genome encodes a second copy of *NPC1* in chromosome 7 (*CmNPC1-C7*), highly similar. Functional assays revealed that the interaction domain of CmNPC1-C7 also interacts with CMV MP, suggesting that both genes could have a role in CMV infection. This study represents the first report linking NPC1 to the infection process of a plant virus, expanding our understanding of plant–virus interactions.

## 1. Introduction

Plant viruses are obligate intracellular parasites that must replicate and navigate the unique cellular architecture of plants. To complete their life cycle, plant viruses must overcome several barriers within the host plant. This includes entry into host cells, translation of their genomes, replication, assembly of new virions, movement from cell to cell through plasmodesmata, and long-distance transport through the plant’s vascular system to develop a systemic infection [1,2]. Given their limited coding capacity, plant viruses completely rely on the host’s cellular machinery to perform these essential functions [3] and, immediately after entry, they perturb the cell metabolism in their own benefit [4,5,6].

Viral replication and intracellular movement are coupled processes that need to be completed to reach plasmodesmata (PDs). Immediately after entry, RNA viruses alter the endomembrane system for their replication to form viral replication complexes (VRCs), which provide a safe environment to protect the viral genome from cellular defense processes like RNA silencing. VRCs contain the viral RNA, viral replication proteins and the necessary host factors [7,8,9]. In many cases, while replicating viral RNAs, VRCs can also move towards PDs [10]. Intracellular trafficking also involves the movement of proteins between membranes in vesicles through the secretory pathway, starting from the Endoplasmic Reticulum (ER), where they are translated, to the Golgi Apparatus (GA) for protein maturation, and from here, to early endosomes (EE) and to late endosomes (LE), also called multi vesicular bodies (MVS), for their distribution to different cell compartments, such as the vacuole, plasma membrane, chloroplasts or mitochondria [11]. For these processes, there are a huge number of host proteins involved, belonging to different protein complexes. This is the case for Melon Necrotic spot virus, which requires an active secretory pathway for its movement to the cell periphery [12], Turnip mosaic virus, which co-opts the Vacuolar sorting receptor VSR4 to promote its replication [13], *Bamboo mosaic virus*, which co-opts an ER luminal-binding and calreticulin to promote its intracellular movement [14] or *Cucumber mosaic virus* (CMV), which requires a Vacuolar Protein Sorting 41 to facilitate viral phloem entry [15]. For a thorough review, see [16]. Proteins involved in those trafficking events can be targets for developing virus-resistant plants.

*Cucumber mosaic virus* is a positive-stranded RNA virus with the widest host range, able to infect over 1200 plant species, including important crops [17]. CMV has a segmented genome consisting of three RNAs: RNA1 encodes a replicase protein (1a), RNA2 encodes a polymerase (2a) and a silencing suppressor protein (2b), whereas RNA3 is devoted to virus movement and encodes the movement protein (MP) and the coat protein (CP) from two separate subgenomic RNAs [18]. CMV has a huge number of strains, which are divided into two subgroups (SG): SG I, whose model strain is FNY, and SG II, whose model strain is LS and that display about 70% homology in their sequence [18]. The ability of CMV to infect a wide range of hosts is partly due to its genetic flexibility and the multifunctional nature of its proteins, which interact with various host factors to facilitate different stages of the viral life cycle [19]. Resistance to CMV is usually complex and recessive, with several genes involved. In melon (*Cucumis melo* L.) CMV resistance involves several genes [20,21], with *cmv1*, the major resistance gene encoding a Vacuolar Protein Sorting 41 (VPS41) [15]. VPS41 is the catalytic subunit of the “homotypic fusion and vacuole protein sorting” (HOPS) complex that plays an important role in the intracellular trafficking of proteins from the GA, through LE to the vacuole [22]. Two independent mutations in CmVPS41, G85E and L348R, were identified to cause the resistance in different exotic melon accessions [15,23]. Mutant CmVPS41s restrict the systemic movement of CMV by blocking its movement in bundle sheath (BS) cells, thereby preventing phloem entry and long-distance transport. This restriction in the BS cells is dependent on the viral MP [24,25]. Moreover, CmVPS41 localizes to the LE and plasma membrane and develops transvacuolar strands (TVS) that correlate with the susceptibility, since mutant CmVPS41s are unable to develop them. TVS are thought to allow CMV to go through the vacuole to reach the PDs of the BS cells to invade phloem cells [26].

The CMV movement protein (MP) is the virulence factor for CMV infection in melon, determined by the CmVPS41 [24]. Viral MPs participate in many of the trafficking events mentioned above, being specialized proteins evolved by plant viruses that facilitate cell-to-cell virus movement and modify plasmodesmata to allow the passage of viral ribonucleoprotein complexes or virions. Consequently, MPs can interact with many host factors to allow intra- and intercellular viral movement [27]. Thus, here we have investigated other host factors that could interact with CMV MP and participate in the same pathway to allow CMV transport towards or through the PD. To address this, we have generated a CMV-infected melon yeast two-hybrid (Y2H) library and screened for potential host targets of CMV MP. This approach led to the identification of a Niemann-Pick C1 (NPC1) protein homolog—a sterol transporter previously implicated in the infection mechanisms of several animal viruses [28].

## 2. Materials and Methods

### 2.1. Plant Material, Viral Strains and Yeast Strains

Melon (*Cucumis melo* L.) cultivar Piel de Sapo (PS) was used for Y2H library preparation. Melon seeds were treated with 2.5 g/L Merpan (ADAMA essentials, Madrid, Spain) for 5 min, rinsed thoroughly and soaked in water overnight. Seeds were pre-germinated for around 3 days in wet plates at 28 °C with a photoperiod of 12 h under light and 12 h in the dark. The seedlings were planted in a fitotron (Fitotronic Version 2, Inkoa, Erandio, Spain) under long day conditions consisting of 22 °C for 16 h with light and 18 °C for 8 h in the dark throughout the whole infection. Zucchini squash (*Cucurbita pepo* L.) Chapin F1 (Semillas Fitó SA, Barcelona, Spain) served as host to generate viral inocula for melon infections, and it was grown in a growth chamber SANYO MLR-350H (Sanyo Electric Co., Osaka, Japan) in long-day conditions consisting of 22 °C for 16 h and 18 °C for 8 h in the dark for all infections. Viral inoculum was prepared by grinding the new leaves of infected zucchini squash in 0.2% diethyl dithiocarbamate of sodium (pH = 7.1–7.2) buffer (DIECA) in the presence of active carbon to disrupt the cells more efficiently. Cotyledons of 7 to 10-day-old melon plants were rub-inoculated with the viral sap. For agroinfiltration, *N. benthamiana* plants were grown in the greenhouse in long-day conditions consisting of 24–28 °C with light for 16 h and 22–24 °C for 8 h in the dark.

CMV strain FNY was used as viral inoculum to prepare the material for the Y2H library. *Saccharomyces cerevisiae* (*S. cerevisiae*) strains Y2HGold and Y187 (Takara Bio Europe AB, Göteborg, Sweden) were used for Y2H screening and one-by-one assays.

### 2.2. Plasmid Construction

For the Y2H assays, to generate the pGBKT7-MP FNY (for library screening) and the pGBKT7-MP LS constructs (for one-by-one assays), the CMV-MP coding sequences were PCR amplified using specific primers (see Supplementary Table S1) and cloned into the pENTR/D-TOPO plasmid (Thermo Scientific, Thermo Fisher Scientific, Vilnius, Lithuania). Then, the PCR products were inserted into the pGBKT7 vector (Takara Bio Europe AB, Göteborg, Sweden) at the *BamHI* and *EcoRI *(New England Biolabs, Ipswich, MA, USA) sites using T4 DNA ligase (Thermo Scientific, Thermo Fisher Scientific, Vilnius, Lithuania) according to the manufacturer’s instructions. The pGBKT7-CmVPS41 PS and pGBKT7-CmVPS41 SC were previously generated [26].

For the bimolecular fluorescence complementation (BiFC) assay, the coding sequences of the interaction domain (ID) of *C. melo* Niemann-Pick C1 protein-like (MELO3C013507.2.1 and MELO3C017027.2) and a known interactor of CMV-MP, *C. melo* Ascorbate Oxidase 4 (CmAO4), were PCR-amplified using specific primers (see Supplementary Table S1). The PCR products were first cloned into the entry vector pBSDONR P1-P4 [29] via BP reactions, and subsequently into the dexamethasone-inducible expression vector pBAV154 [30] as N-terminal fusions with partial N-terminal YFP using three-way Gateway LR reactions with MultiSite Gateway^®^ Pro (Invitrogen, Thermo Fisher Scientific, Lithuania). This resulted in the constructs CmNPC1 ID-C11:nYFP, CmNPC1 ID-C7:nYFP and CmAO:nYFP. Mutant CmNPC1 ID constructs were cloned in the same way, resulting in all of them as N-terminal fusions with partial N-terminal YFP (CmNPC1 ID-C11#1, 2, 3, 4 or 5:nYFP). Constructs MP FNY:cYFP and MP LS:cYFP encoding the MPs from CMV-FNY or CMV-LS fused to the C-terminal YFP fragment (cYFP) were previously generated in [26]. All constructs were verified by sequencing with the Sanger method using an ABI 3730 DNA Analyzer (Applied Biosystems, Carlsbad, CA, USA) for capillary electrophoresis and fluorescent dye terminator detection. Correct insertion and orientation of all constructs were verified with Sequencher^®^ version 5.0 sequence analysis software (Gene Codes Corporation, Ann Arbor, MI, USA; http://www.genecodes.com). Correct plasmids were transformed either into *Agrobacterium tumefaciens* GV3101 for transient expression experiments in *N. benthamiana* or into *S. cerevisiae* strains for Y2H assays.

### 2.3. RNA Isolation

Total RNA was obtained with Spectrum™ Plant Total RNA Kit (Sigma, Saint Louis, MO, USA) following the manufacturer’s instructions. Quality and quantity of RNA were determined by a 1% agarose gel electrophoresis and NanoDrop™ ND-1000 Spectrophotometers (Thermo Fisher Scientific, Waltham, MA, USA). Total RNA was treated with DNase I (Takara, Kyoto, Japan) to eliminate contaminating genomic DNA.

### 2.4. Library Construction

The Y2H library was constructed using the “Make Your Own ‘Mate & Plate” Library System (Takara Bio Europe AB, Göteborg, Sweden), following the manufacturer’s instructions. Briefly, cDNA was synthesized from total RNA using the SMART™ cDNA Library Construction Kit (Takara Bio Europe AB, Göteborg, Sweden) primed with the CDSIII/6 random primer, and the resulting cDNA was subsequently amplified through long-distance PCR (LD-PCR). The amplified cDNA was then normalized utilizing CHROMA SPIN™ + TE-400 Columns (Takara Bio Europe AB, Göteborg, Sweden), following the manufacturer’s protocol. The normalized cDNA was co-transformed with a linearized pGADT7-Rec vector into yeast strain Y187 via high-efficiency transformation after size and quality check. Transformed cells were plated on synthetic dropout medium lacking leucine (SD/-Leu; Minimal SD Base 26.7 g/L, dropout mix-Leu 0.69 g/L, agar 2%, kanamycin 50 µg/mL) and incubated at 30 °C for 4–5 days. Finally, the library was titrated, reaching 1.2 × 10^9^ independent clones. Yeast colonies were harvested in a freezing medium composed of 25% glycerol in YPDA broth, aliquoted, flash-frozen in liquid nitrogen, and stored at −80 °C until further use. A 1 mL aliquot of this library was used for subsequent screening using the Matchmaker Gold Yeast Two-Hybrid System (Takara Bio Europe AB, Göteborg, Sweden).

### 2.5. Bait Vector pGBKT7 Testing and Protein Expression

To ensure the bait construct was suitable for screening, the pGBKT7 vector containing the MP gene was transformed into yeast strain Y2HGold and the transformed cells were selected on SD/-Trp agar plates. Toxicity and autoactivation of the bait were assessed by monitoring growth and colorimetric changes on SD/-Trp and SD/-Trp/X-α-Gal/AbA plates (Takara Bio Europe AB, Göteborg, Sweden. Protein expression of the MP was verified by Western blot with extracts prepared from yeast cultures using the rapid alkaline extraction method [31]. Briefly, yeast cells were treated with 0.1 M NaOH for 5 min at room temperature. The cells were then pelleted, resuspended directly in SDS-PAGE sample loading buffer, and boiled for 3 min to release the proteins. The protein extract was then probed with an anti-MP antibody (1:5000 dilution).

### 2.6. Y2H Screening of Proteins Interacting with CMV-FNY MP

A single large colony (2–3 mm) of Y2HGold yeast containing the bait vector pGBKT7-MP FNY was used to prepare a concentrated overnight culture in SD/-Trp medium, achieving a cell density of >1 × 10^8^ cells/mL. The bait culture was mixed with a 1 mL aliquot of prey library in Y187 yeast (>2 × 10^7^ cells), and the mixture was incubated overnight to facilitate mating. After 20 h, zygote formation was confirmed via epifluorescence microscopy. The mated culture was centrifuged, resuspended, and plated onto viability (SD/-Trp, SD/-Leu, SD/-Trp/-Leu) and selective (SD/-Trp/-Leu/X-α-Gal/AbA) media. After 3 to 5 days of incubation at 30 °C, viability plates were assessed, confirming that over 1 million diploid cells were screened. Blue colonies on the selective plates were further validated by transference to more stringent media (SD/-Ade/-His/-Trp/-Leu/X-α-Gal/AbA). Positive colonies, indicating potential interacting proteins, were isolated for further analysis. Plasmids from these positive colonies were extracted using the Easy Yeast Plasmid Isolation Kit (Takara Bio Europe AB, Göteborg, Sweden. The inserts were sequenced using CMV-2F and CMV-2R primers and analyzed via BLAST (v2.9.0) against the Melonomics database (https://www.melonomics.net/melonomics.html#/blast, 18 March 2019).

### 2.7. One-by-One Y2H

Yeast strains, carrying either pGADT7:prey or pGBKT7:bait constructs, were co-cultured in 1XYPDA medium with kanamycin (50 µg/mL) at 30 °C for 20 h. Following incubation, 100 µL of the culture was plated on SD/-Trp/-Leu/X-α-Gal agar and incubated for 3–5 days at 30 °C. Blue colonies were transferred to increasingly restrictive media (SD/-Trp/-Leu/X-α-Gal/AbA, followed by SD/-Trp/-Leu/X-α-Gal/AbA/-Ade/-His). Colonies that remained blue on the most restrictive medium were identified as true interactors.

### 2.8. Transient Expression in Nicotiana benthamiana

*A. tumefaciens* strains harboring the appropriate constructs were grown at 28 °C with selective antibiotics for 24–48 h. After centrifugation, the bacterial pellet was resuspended in induction buffer (10 mM MgCl_2_ and 0.15 mM acetosyringone) to a final optical density (OD600) of 0.4. The suspension was then incubated in the dark for 2 h to induce expression. For coinfiltration of multiple plasmids, the suspensions were combined in equal volumes and introduced into the abaxial surfaces of the 3rd or 4th mature leaves of 4-week-old *N. benthamiana* plants using a syringe without a needle. For constructs derived from the pBAV154 vector, which allows dexamethasone-inducible expression, 24 h after agroinfiltration, a 50 µM dexamethasone solution (Sigma-Aldrich, Saint Louis, MO, USA) was applied using a brush to the adaxial side of the infiltrated leaf to induce expression. Fluorescence expression was then observed 20 h following dexamethasone application for the pBAV154-derived constructs, and 48 h post-agroinfiltration for constructs driven by the 35S promoter.

### 2.9. Confocal Laser Scanning Microscopy

Images were acquired using a Leica TCS-SP5 II confocal microscope (Leica Microsystems, Exton, PA, USA) using a 63× water immersion objective (NA 1.2) and a zoom factor of 1.6. In BiFC assays, YFP was excited with a blue argon ion laser (514 nm), and fluorescence was detected between 530 and 630 nm using a HyD detector. Image analysis was performed with Fiji software (version 1.52i).

### 2.10. Co-Immunoprecipitation (Co-IP)

*N. benthamiana* leaves were agroinfiltrated with the appropriate constructs and samples were collected after four days. Protein extracts from cells co-expressing MP FNY-myc and CmNPC1-Chr11 ID:GFP or CmNPC1-Chr7 ID:GFP were prepared following standard procedures [32] and subjected to immunoprecipitation (IP) using an anti-GFP antibody (Thermo Fisher Scientific, Waltham, MA, USA). Western blot analysis was performed using anti-myc (1:2000) antibody (Thermo Fisher Scientific, Waltham, MA, USA) to detect MP FNY:myc and anti-GFP (1:2000) to detect both CmNPC1 IDs:GFP. Secondary antibodies used were anti-mouse and anti-rabbit (1:20,000) (Thermo Fisher Scientific, Waltham, MA, USA), respectively.

### 2.11. Bioinformatic Analysis of CmNPC1

The *CmNPC1-C11* and *CmNPC1-C7* sequences were obtained from the Melonomics database (https://www.melonomics.net/, accessed on 5 May 2019) [33] and aligned using Clustal Omega (https://www.ebi.ac.uk/jdispatcher/msa/clustalo, accessed on 5 May 2019) [34] and translated using the ExPASy translation tool (https://web.expasy.org/translate/, accessed on 5 May 2019) [35]. Additionally, a sequence logo was generated to visually represent the aligned sequences using the tool available at https://weblogo.berkeley.edu (accessed on 15 January 2026). The amino acid sequences of the two different CmNPC1 genes were used to predict their three-dimensional protein structures. Structural models were generated using the AlphaFold 3 platform (https://alphafoldserver.com, accessed on 30 January 2026), Ref. [36]) to compare the protein variants. The resulting models, along with their associated predicted aligned error (PAE) plots, were analyzed to assess model confidence and to visualize key domains, including the sterol-sensing domain and the putative interaction domain.

### 2.12. *Structural modeling and interfacial analysis*

Structural model of the *C. melo* NPC1-C11 in complex with CMV FNY MP was generated using the AlphaFold3 server [36]. The full-length amino acid sequence was used as input for heteromeric assembly with a 1:1 stoichiometry. Model confidence was assessed using the predicted local distance difference test (pLDDT), predicted alignment error (PAE), and interface predicted template modeling (ipTM) scores. Structural analysis, interface mapping, and high-resolution visualization were performed in UCSF ChimeraX (v1.8).

## 3. Results

### 3.1. Screening of a Melon cDNA Library Against CMV Movement Protein

A normalized cDNA library was built from RNA extracted from CMV FNY-infected melon PS as indicated in Materials and Methods. To identify melon host factors involved in CMV infection, the library was used as a prey and screened with full-length CMV-FNY MP as a bait in the Gal4 DNA-binding domain vector. Following mating and selection on SD-Trp/-Leu/X-α-Gal/Aba plates, 30 blue colonies were identified on the most selective media (SD/-Trp/-Leu/X-α-Gal/AbA/-His/-Ade). cDNA from these colonies was sequenced and compared to the *C. melo* CM3.6.1 reference genome [33], revealing five potential interactors each with different lengths in the interacting domains and represented one to three times in the surviving colonies, except one of them, a Niemann-Pick C1, which was represented in 22 independent clones, carrying all of them the same 1011 bp length fragment of the gene (Table 1).

### 3.2. A Niemman’s Pick C1 Protein Interacts with CMV-MP

**Figure 1:**
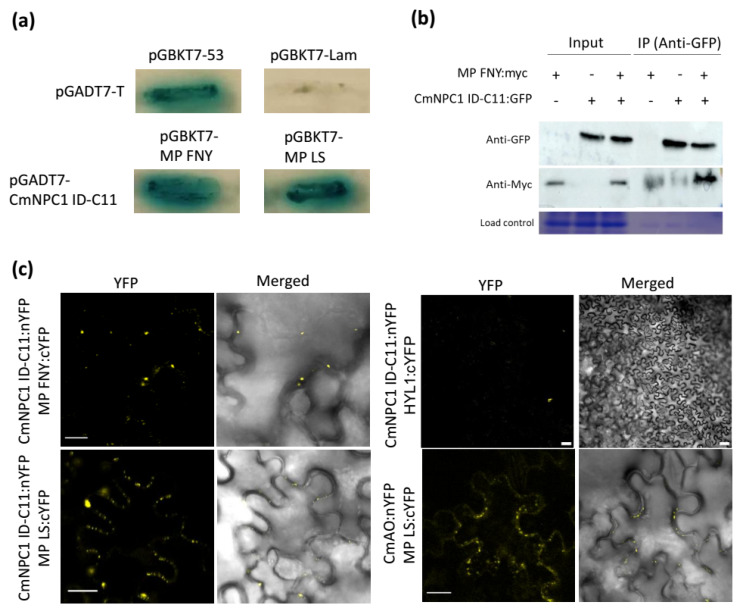
Interaction between CmNPC1 ID-C11 and CMV MPs. (**a**) Yeast-two-hybrid (Y2H) assay between CmNPC1 ID-C11 and CMV-MPs in SD-Trp/-Leu/X-alpha-Gal/AbA/-His/-Ade agar plates. Each cell shows the results of the Y2H interaction combination of prey in vector pGADT7 per bait in vector pGBKT7. Growth of a strong or light blue colony indicates interaction between prey and bait, whilst the absence of growth or white colonies indicates no interaction. Controls are pGADT7-T (prey) in combination with either bait pGBKT7-53 (positive interaction) or bait pGBKT7-Lam (no interaction). (**b**) Co-immunoprecipitation of MP FNY:myc with CmNPC1 ID-C11:GFP. Protein extracts from cells co-expressing MP FNY:myc and CmNPC1 ID-C11:GFP were subjected to immunoprecipitation (IP) using an anti-GFP antibody. Western blot analysis was performed using anti-myc (1:2000) to detect MP FNY:myc and anti-GFP (1:2000) to detect CmNPC1 ID-C11:GFP. Secondary antibodies used were anti-mouse and anti-rabbit (1:20,000), respectively. Coomassie staining was used as a loading control. (**c**) In planta BiFC assay between CMV MPs and CmNPC1 ID-C11. CmAO:nYFP, *C. melo* L-ascorbate oxidase used as positive MP interacting control, and HYL1:cYFP used as negative CmNPC1 interacting control. “Merged”: YFP and bright field channel together. Images are representative of three leaves in three independent experiments. BiFC scale bars = 20 μm length.

To further validate these interactors, one-by-one Y2H experiments were carried out, testing each individual clone against MP from CMV-LS and from CMV-FNY. Out of the five interactors identified during the screening, only the Niemann-Pick C1 protein-like (MELO3C013507.2.1), which maps in chromosome 11 (CmNPC1-C11) (Figure 1a), and a 5′-Ribose Phosphate Isomerase (5RP) (MELO3C005310.2.1) were confirmed, whereas none of the other candidate interactors, nor the negative control (pGBKT7-Lam with pGADT7-T) showed any interaction (Figure 1a). As CmNPC1-C11 was highly represented among the clones and given its relevance in the infection of several viruses in human cells, we focused on this protein. The interaction between CmNPC1-C11 interactor domain (CmNPC1 ID-C11) and CMV-MP was then confirmed by bimolecular fluorescence (BiFC) and Co-IP assays (Figure 1b,c). For Co-IP, CMV-MP was myc-tagged (MP FNY:myc), and CmNPC1 ID-C11 was GFP-tagged (CmNPC1 ID-C11:GFP). Pull-down was carried out using anti-GFP antibody, and a band corresponding to CMV-MP was developed with anti-myc (Figure 1b). For BiFC, CmNPC1 ID-C11 fused to the N-terminus of Yellow Fluorescent Protein (YFP) (CmNPC1 ID:nYFP) was co-expressed with MP FNY fused to the C-terminus of YFP (MP FNY:cYFP) in *N. benthamiana* leaves. After dexamethasone induction in the agroinfiltrated leaves, reconstitution of YFP was observed as discrete spots around the cell, confirming the interaction (Figure 1c). To determine the specificity of this interaction, the BiFC assay was repeated with MP from the CMV-LS strain. As shown in Figure 1c, MP LS also interacted with CmNPC1 ID-C11, demonstrating that CmNPC1-C11 interacts with MPs from both subgroup I (FNY) and subgroup II (LS) strains of CMV. As a positive control, the *C. melo* Ascorbate Oxidase 4 fused to the N-terminal YFP (CmAO4:nYFP) was used. CmAO4 is known to interact with CMV-SG MP, an SG II CMV strain [37]. HYL1, a protein involved in the biogenesis of miRNAs, was used as a negative control for CmNPC1 interaction [38]. Additionally, control experiments using another non-related protein as AGO1, involved in post-transcriptional RNA silencing [39], showed no YFP signal with CMV MPs (Supplementary Figure S1).

### 3.3. Structure of the CmNPC1 ID-C11

**Figure 2:**
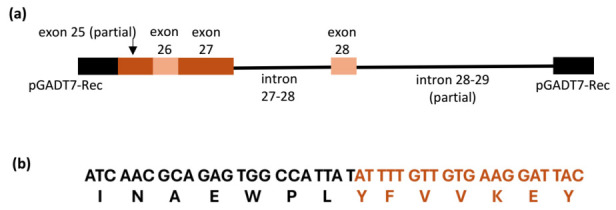
(**a**). Structure of the CmNPC1 ID-C11. Pale and dark red boxes represent exons, black lines connecting exons represent introns, and black boxes represent the pGADT7 vector. (**b**) Sequence of the in-frame cloning of CmNPC1 ID in the Y2H vector: in black, sequence from pGADT7-Rec vector, in red, partial CmNPC1-C11 exon 25, starting from nucleotide 50 within the exon.

To define the interaction domain of CmNPC1-C11, we aligned the sequences of the inserts obtained from yeast two-hybrid (Y2H) screened colonies against the melon genome. Notably, all colonies mapped to the same region of CmNPC1, suggesting a consistent interaction domain. The alignment results indicated that the CmNPC1 ID starts at position 50 of exon 25, includes spliced whole exons 26 and 27, followed by intron 27–28, exon 28, and the first 442 base pairs of intron 28–29 (Figure 2a). All colonies started with the same sequence within exon 25 (ATTTTGTT), which lies in frame with the Smart III ORF in the pGADT7 vector and guarantees the in-frame expression of the interacting peptide (Figure 2b, Supplementary Figure S2A). Interestingly, the 3′end from all colonies contains a Poly A-like sequence of at least 22 Adenosine residues (Supplementary Figure S2B). Additionally, RT-PCR experiments aimed at amplifying the intermediary unspliced forms using primers in exon 25 and exon 28 detected the 317 bp band corresponding to the completely spliced RNA but also the 569 bp band corresponding to the form including unspliced 27–28 intron (Supplementary Figure S3), indicating that at least intron 27–28 was present in the RNA pool of both infected and non-infected plants. Altogether, the structure of the interaction domain would suggest that true uncommon partially unspliced mRNAs are prematurely finished, and polyadenylated mRNA exists in the cell pool and that this genetic arrangement would be necessary for the interaction with MP. This experiment would also suggest that neither the unspliced nor the spliced form would be overexpressed upon CMV infection.

### 3.4. Introns and Exon 28 Are Dispensable for the Interaction with CMV-MPs

**Figure 3:**
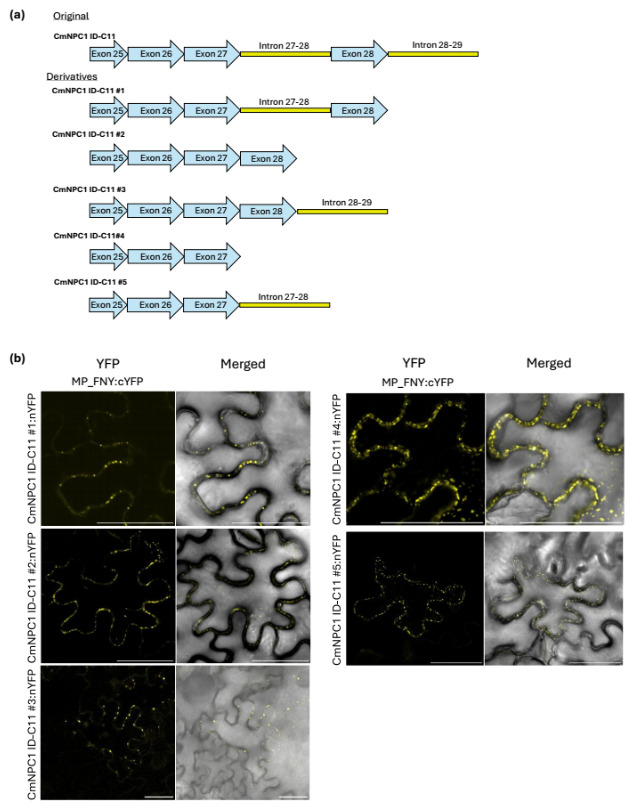
(**a**) Graphical representation of the exonic and intronic composition of the different constructs derived from the original *CmNPC1 ID-C11*. (**b**) BiFC assays in leaf epidermal cells showing interaction between NPC1 ID mutants #1 to #5:nYFP and MP FNY:cYFP. “Merged”: YFP and bright field channels together. Images are representative of three leaves in three independent experiments. Scale bar = 50 µm.

In the Y2H interacting clones, the unspliced intron 27–28 carries six in-frame stop codons (the first one only three nucleotides inside the intron), which would impede the correct translation of the whole putative interacting peptide (Supplementary Figure S4). To understand more precisely the putative role of the introns in the interaction and map the regions of the ID needed for the interaction with CMV MPs, several successive deletions were carried out in the original clone, generating five constructs (Figure 3a). In construct 1, intron 28–29 was removed. In construct 2, both introns were removed, leaving a fully spliced construct with only exons. In construct 3, intron 27–28 was removed, keeping intron 28–29. Construct 4 carried only the spliced exons 25, 26 and 27, missing exon 28. Finally, construct 5 carried exons 25, 26, 27 and intron 27–28, missing exon 28 and intron 28–29. When tested by BiFC, all constructs displayed a positive interaction with CMV-MP from both FNY and LS strains, indicating that the introns, despite having been conserved and cloned 22 times, have no role in the interaction with CMV MPs (Figure 3b and Supplementary Figure S5). Moreover, the results indicated that exon 28 was also dispensable for the interaction, leaving only partial exon 25 and exons 26 and 27 as a core interaction domain (CID) that would generate a putative peptide of a maximum of 82 amino acids, carrying the necessary residues for the interaction with CMV MP.

### 3.5. Structure of the Melon NPC1 Gene

**Figure 4:**
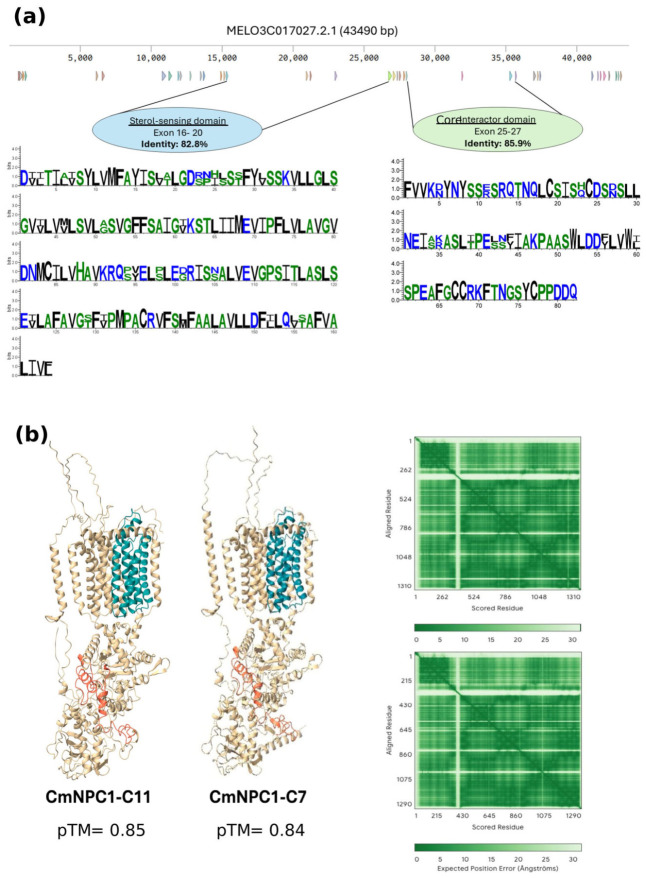
Comparison between CmNPC1s. (**a**) Structure of CmNPC1-11 (MELO3C013507.2.1) gene showing exons as arrowheads, and comparison between SSD and CID from CmNPC1-C11 and CmNPC1-C7 proteins. The larger the amino acid letter, the higher the level of conservation. (**b**) Structural models of CmNPC1-C7 and CmNPC1-C11. Left and center panels show the two CmNPC1 predicted structures with the sterol-sensing domain (cyan) and the putative interaction domain (red). Right panels display the predicted aligned error (PAE) plots indicating model confidence across residue pairs, with lower values in darker green representing higher confidence predicted by AlphaFold. pTM: Predicted template modeling scores.

The NPC1 ID identified by Y2H mapped to chromosome 11. The native *CmNPC1-C11* gene (MELO3C013507.2) is 43,490 bp long, with 39 exons and a final coding sequence of 3906 bp, which is translated into a transmembrane protein with 13 transmembrane domains. NPC1 is a sterol transporter carrying a conserved sterol-sensing domain (SSD) [40]. However, a deeper look into the melon genome allowed us to find a second *CmNPC1* gene in chromosome 7, *CmNPC1-C7* (MELO3C017027.2), whose ID was not found among the NPC1 clones in the Y2H screening. This second CmNPC1 gene was 15,239 bp long, with 39 exons and a final coding sequence of 3882 bp. Thus, the final coding sequence is very similar to that of *CmNPC1-C11*, with the difference in genomic length due to much shorter introns in the *CmNPC1-C7* gene. The coding sequences show 70.28% identity, whereas the proteins show 68.2% identity and 82.03% similarity, with two regions, the SSD and the CID, showing a higher level of conservation. At the DNA level, the SSD displayed 77% identity, and the CID region showed an 82% (Supplementary Figure S6), which at the protein level increased up to 82.8% in the SSD and 85.90% in the CID (Figure 4a). The structure, as given by the AlphaFold 3 platform (https://alphafoldserver.com, accessed on 30 January 2026), was highly similar, with the SSDs expanding five transmembrane domains and small extramembrane domains and ID mapping in a large extramembrane domain (Figure 4b). This model was also used to determine if the experimentally validated ID remains accessible for viral docking in the full-length folded CmNPC1-C11 protein. The resulting model suggests that the viral MP docks within a concave pocket formed by a large C-terminal extramembrane loop, placing the MP in direct contact with the identified ID between residues 909 and 991. While this docking is consistent with our experimental data, the predicted aligned error (PAE) is high, indicating a low confidence in the exact relative orientation of the complex (Supplementary Figure S7). The two CmNPC1 genes present, however, a quite different expression pattern, according to the Melonet DB expression atlas (https://melonet-db.dna.naro.go.jp/ap/top, accessed on 25 September 2023) [41], with that of *CmNPC1-C11* being three times higher than *CmNPC1-C7*. Moreover, at the tissue level, both genes showed a different expression pattern, with *CmNPC1-C11* being more expressed in mature fruit, leaves and roots, and *CmNPC1-C7* reaching its highest levels in petals and roots (Supplementary Figure S8).

### 3.6. CmNPC1-C7 Interacts with CMV MP

**Figure 5:**
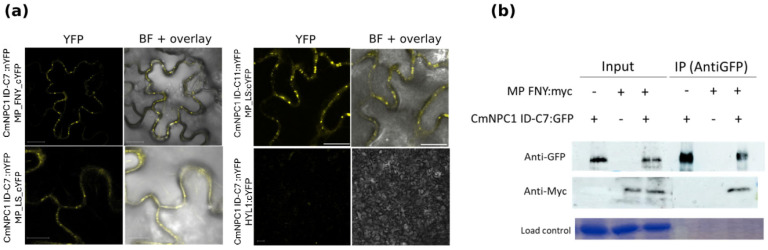
Interaction between CmNPC1 ID-C7:nYFP and CMV MPs. (**a**) BiFC assays in leaf epidermal cells showing interaction between CmNPC1 ID-C7:nYFP CMV LS/FNY:cYFP. Representative image from three leaves in three independent experiments. CmNPC1 ID-C11 was used as a positive interacting control, and HYL1:cYFP was used as a negative CmNPC1 ID-C7 interacting control. “Merged”: YFP and bright field channel together. Scale bar = 50 µm. (**b**) Co-immunoprecipitation of MP-FNY:myc with CmNPC1 ID-C7:GFP. Coomassie staining was used as a loading control. Protein extracts from cells co-expressing MP FNY:myc and CmNPC1 ID-C7:GFP were subjected to immunoprecipitation (IP) using an anti-GFP antibody. Western blot analysis was performed using anti-myc (1:2000) to detect MP FNY:myc and anti-GFP (1:2000) to detect CmNPC1 ID-C7:GFP. Secondary antibodies used were anti-mouse and anti-rabbit (1:20,000), respectively. Coomassie staining was used as a loading control.

To test the specificity of the interaction with CMV MP, the interactor domain of *CmNPC1-C7* was identified by expanding the same interval from exon 25 to exon 27 and cloned into the BiFC vectors fused to the N-terminus of YFP. After coinfiltration with MP fused to cYFP into *N. benthamiana* plants, the same yellow spots as those previously found with CmNPC1 ID-C11 were detected, demonstrating that CmNPC1 ID-C7 could also interact with both CMV MPs (Figure 5a). The interaction was further validated through Co-IP experiments (Figure 5b). Thus, both CmNPC1 proteins are able to interact with CMV MP, suggesting that both could have a role in the infection by CMV.

## 4. Discussion

In this study, we successfully generated and validated a melon cDNA library from *Cucumis melo* PS CMV-infected leaves, which enabled us to screen for host proteins interacting with CMV-MP. Through this screening, we have identified a Niemann-Pick C1 protein homolog that interacts with CMV MP, a viral protein involved in the cell-to-cell transport of the virus. In the Y2H screening, we found up to 22 yeast clones carrying the same fragment of a *CmNPC1* gene. All clones were partially spliced and contained two introns, suggesting that they could somehow have been generated by alternative splicing to translate a protein with alternative epitopes that could bind the viral MP to lead the viral movement. Alternative splicing is common in nature and in response to pathogens, contributing to the diversification and adaptation of biological processes [42]. In fact, RT-PCR experiments aimed at amplifying the intermediary unspliced forms confirmed the presence of at least intron 27–28, together with the fully spliced form, in the RNA pool of both infected and non-infected plants (Supplementary Figure S3). However, there were six stop codons in the correct frame in the first intron, which would prevent the translation of an alternative CmNPC1 protein (Supplementary Figure S4). In fact, the elimination of those introns and even exon 28 maintained the binding capacity to MP, indicating that the introns and exon 28 are dispensable for the interaction with CMV MP. Alternatively, introns and exon 28 could be necessary for interaction in yeast and not in the plant. Thus, if the introns had any role in CMV infection, it would not be at the interaction step.

In melon, we have identified two highly homologous *NPC1* genes, both with distinctive conserved SSD and ID, and both IDs capable of interacting with CMV MP. However, the two genes exhibit markedly different expression patterns and tissue distributions. All 22 clones found in the Y2H screening mapped exclusively to *CmNPC1-C11*, whose expression is three times higher than that of NPC1-C7 according to the Melonet DB expression atlas (https://melonet-db.dna.affrc.go.jp/ap/mvw, accessed on 25 September 2023). In line with this difference in expression, and considering that the cDNA library was normalized, we would have expected to find roughly five clones belonging to *CmNPC1-C7*. However, this is not the case, suggesting that, despite its interaction with CMV-MP, *CmNPC1-C7* would have a less significant role in the viral infection than its expression level would imply.

NPC1 is a conserved transmembrane protein involved in cholesterol trafficking in animals between the LE and lysosome membranes in the cell through its sterol-sensing domain [43]. It has a role in intracellular trafficking in yeast as well, where the NPC1 homolog Ncr1p is vital for sorting vacuolar proteins and is functionally linked to the broader Vacuolar Protein Sorting (VPS) pathway [44]. Interestingly, it is also involved in several viral infections. The protein NPC1 is the receptor of Ebolavirus (EBV) and other filoviruses, through their glycoprotein, promoting viral internalization into the cell [45,46]. Likewise, NPC1 is also involved in Hepatitis C and Hepatitis A viruses’ infection [44], where its role in maintaining membrane integrity through its cholesterol transport activity is required for viral replication in the membranous VRC [47,48]. In Reovirus infections, its cholesterol transporter function is required for delivering viruses from LE to the cytoplasm to initiate replication [49]. A deficiency in human NPC1 can lead to reduced viral replication during HIV-1 infection [50] and to a restriction of SARS-CoV 2 entry [51]. Moreover, effective binding of EBV GP to NPC1 necessitates the presence of the already mentioned HOPS complex [28], which includes VPS41, the protein previously identified controlling CMV infection in melon [15]. In our pathosystem, Real et al. [26] showed that CmVPS41 from susceptible melon PS interacts with the MP from CMV FNY strain, but not with the MP from CMV LS. The discovery that CmNPC1 interacts with the MP from both strains suggests a potentially more universal role for CmNPC1 in the CMV infection process, possibly in conjunction with CmVPS41. Thus, the cellular function of VPS41 and NPC1 seems to be tightly linked to promote protein and lipid trafficking in the cell, and they could also play a joint role in CMV infection in melon. A putative interaction between CmVPS41 and CmNPC1 would be demonstrated by using the full-length CmNPC1, which was not possible for this study due to the challenges of cloning this large, transmembrane protein.

In plants, NPC1 has not been extensively studied. Arabidopsis encodes two *NPC1* genes whose mutation leads to altered sphingolipid levels, altered development and reproductive defects in heterozygosity and lethality in homozygosis [52]. However, NPC1 had never been related to plant viruses to date. Sterols are known to alter membrane fluidity by dynamically packing and unpacking lipids depending on environmental conditions, thereby facilitating the movement of proteins along the membrane [53]. This would allow the lateral movement of membrane-bound viral proteins as well, contributing to intracellular viral movement towards the PDs. In this scenario, the sterol transport activity of NPC1 coupled with its interaction with MP may play a pivotal role in CMV movement to PDs. This hypothesis is supported by our BiFC results, which show the interaction appearing as punctate dots at the cell periphery, compatible with PD localization. This observation suggests that the NPC1-MP complex would act at the site of viral cell-to-cell movement. Further investigation is required to elucidate the biological significance of the NPC1-MP interaction and to uncover the precise mechanism by which NPC1 contributes to CMV movement.

## Figures and Tables

**Table 1:** Y2H screening of the CMV-FNY-infected melon cDNA library.

Protein Name	Melonomics CM4.0 ID	Position in CM3.6.1 Reference Genome	Number of Colonies Identified	Length of the Interaction Domain
Cytochrome P450 78A9-like	MELO3C022246.2.1	chr11:32115888- 32117903 (−strand)	2	327 bp
Niemann-Pick C1 protein-like	MELO3C013507.2.1	chr11:16247002- 16290491 (+strand)	22	1011 bp
Ribose-5-phosphate isomerase A	MELO3C005310.2.1	chr09:20361987- 20363522 (−strand)	3	338 bp
Polyneuridine-aldehyde esterase	MELO3C022211.2.1	chr09:229332- 233980 (+strand)	1	533 bp
Gluthation-S-transferase	MELO3C001175.2.1	chr0:24168871- 24169230 (+strand)	2	373 bp

Proteins were identified after blasting the sequences found in the yeast clones. BLAST was performed in the Melonomics website (https://www.melonomics.net/) using the reference genome assembly CM3.6.1 of *Cucumis melo* L.

## Data Availability

Sequences of the CmNPC1-C7 and CmNPC1-C11 are available at https://www.melonomics.net/.

## Supplementary Materials

The following supporting information can be downloaded at: https://www.mdpi.com/article/10.3390/v18050577/s1, Table S1: Primers used in this study, Figure S1: In planta BIFC negative controls, Figure S2: Flaking regions of CmNPC1 interaction domain with CMV-MP, Figure S3: Introns in the RNA pool, Figure S4: In frame stop codons present in intron 27–28 of CmNPC1-C11, Figure S5: Interactions with CMV LS, Figure S6: DNA comparisons between CmNPC1s, Figure S7: Alphafold model of the interaction between CmNPC1-11 and CMV FNYMP, Figure S8: Expression atlas of both *CmNPC1* genes in different melon tissues.
