# Supplementary material for: The Melon Sterol Transporter Niemann-Pick C1 Protein Is a New Interactor of Cucumber mosaic virus Movement Protein
Source: Viruses. 2026 May 20;18(5):577. doi: 10.3390/v18050577 (PMC13211540; doi:10.3390/v18050577)
Supplement: Supplementary file 1 [file viruses-18-00577-s001.zip › Supplementary Figure S1.pdf]

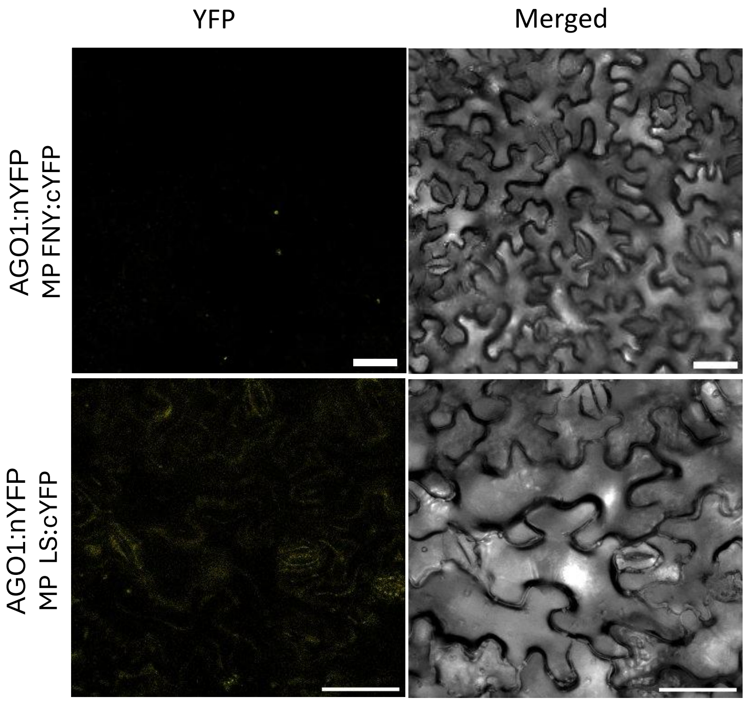

Supplementary Figure S1. In planta BIFC negative controls for CMV MPs lacking interaction with AthAGO1**.** 'Merged': YFP and bright field channels together. BIFC scale bars correspond to 20 µm length.
