# Supplementary material for: The Melon Sterol Transporter Niemann-Pick C1 Protein Is a New Interactor of Cucumber mosaic virus Movement Protein
Source: Viruses. 2026 May 20;18(5):577. doi: 10.3390/v18050577 (PMC13211540; doi:10.3390/v18050577)
Supplement: Supplementary file 1 [file viruses-18-00577-s001.zip › Supplementary Figure S2.pdf]

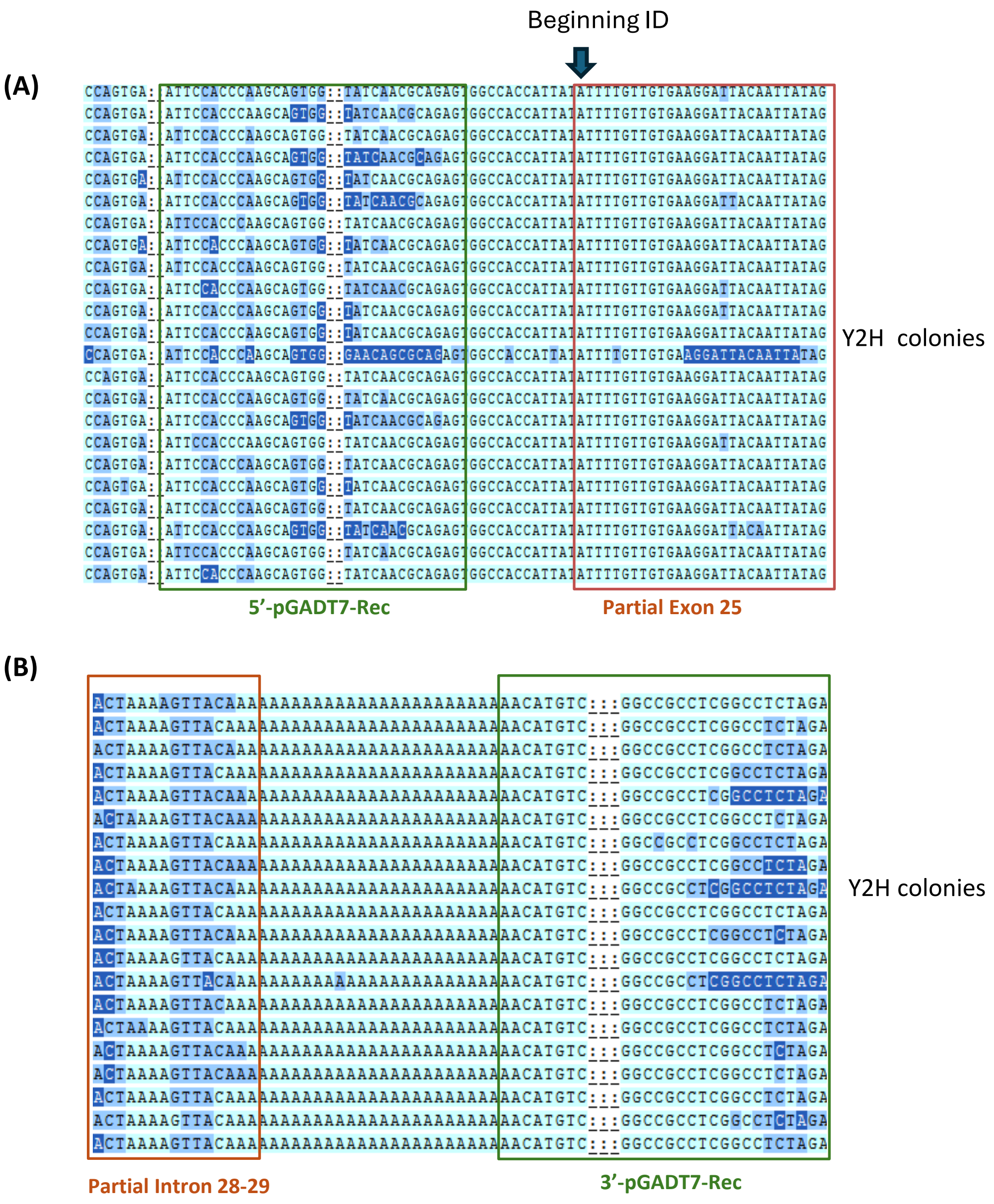

Supplementary Figure S2. Alignment of the flanking regions of all Y2H colonies carrying the *CmNPC1 ID-C11*. (**A**). 5' flanking region from pGADT7-Rec vector and beginning *of CmNPC1 ID-C11* at nucleotide 50 of exon 25. Confidence score of the sequence is indicated in highlighted blue (dark blue corresponds to confidence score <20, medium blue to midrange values and pale blue to confidence score >20, according to Sequencher ® 5 series manual). (**B**). 3' flanking region from sequenced colonies showing the end of partial intron 28-29, a poly A sequence and 3' flanking region of pGADT7-Rec vector.
