# Supplementary material for: The Melon Sterol Transporter Niemann-Pick C1 Protein Is a New Interactor of Cucumber mosaic virus Movement Protein
Source: Viruses. 2026 May 20;18(5):577. doi: 10.3390/v18050577 (PMC13211540; doi:10.3390/v18050577)
Supplement: Supplementary file 1 [file viruses-18-00577-s001.zip › Supplementary Figure S3.pdf]

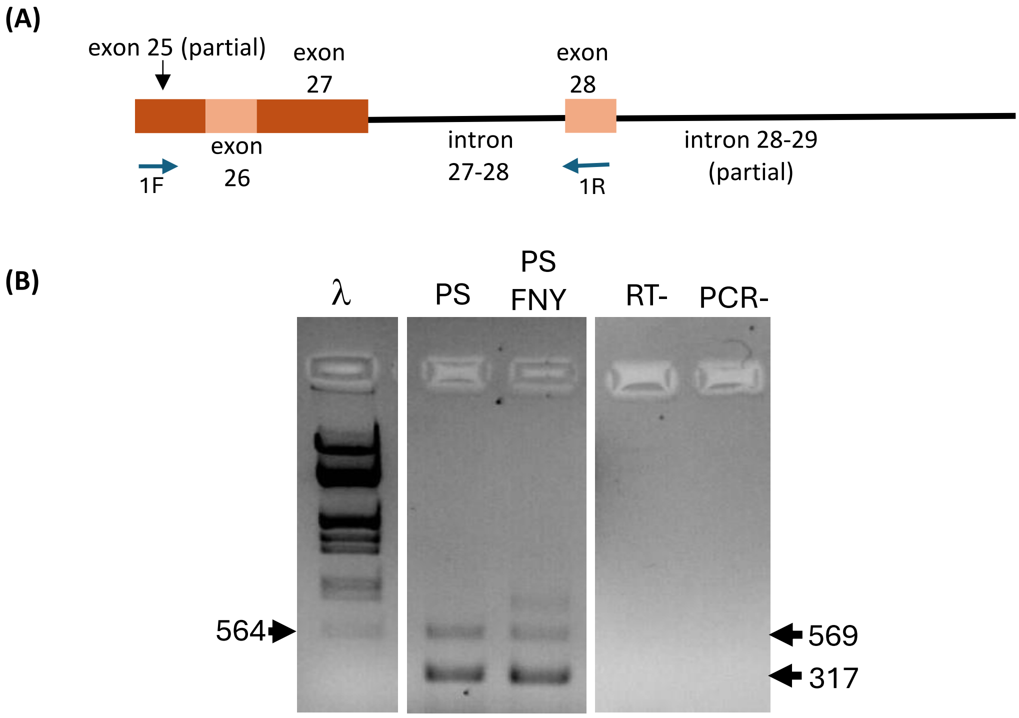

Supplementary Figure S3. Introns in the melon RNA pool**. (A)**. CmNPC1 ID-C11. 1F and 1R are the primers used in RT-PCR to amplify from exon 25 to exon 28. (**B**)**.** RT-PCR. The band at 317 pb corresponds to the fully spliced exons. The 569 bp band corresponds to the fragment retaining the intron 27-28. l: size marker, indicating the 564 bp band. PS: Piel de sapo. PS FNY: FNY-infected PS. RT-: RNA control without reverse transcriptase. PCR-: control without cDNA.
