# Supplementary material for: The Melon Sterol Transporter Niemann-Pick C1 Protein Is a New Interactor of Cucumber mosaic virus Movement Protein
Source: Viruses. 2026 May 20;18(5):577. doi: 10.3390/v18050577 (PMC13211540; doi:10.3390/v18050577)
Supplement: Supplementary file 1 [file viruses-18-00577-s001.zip › Supplementary Figure S5.pdf]

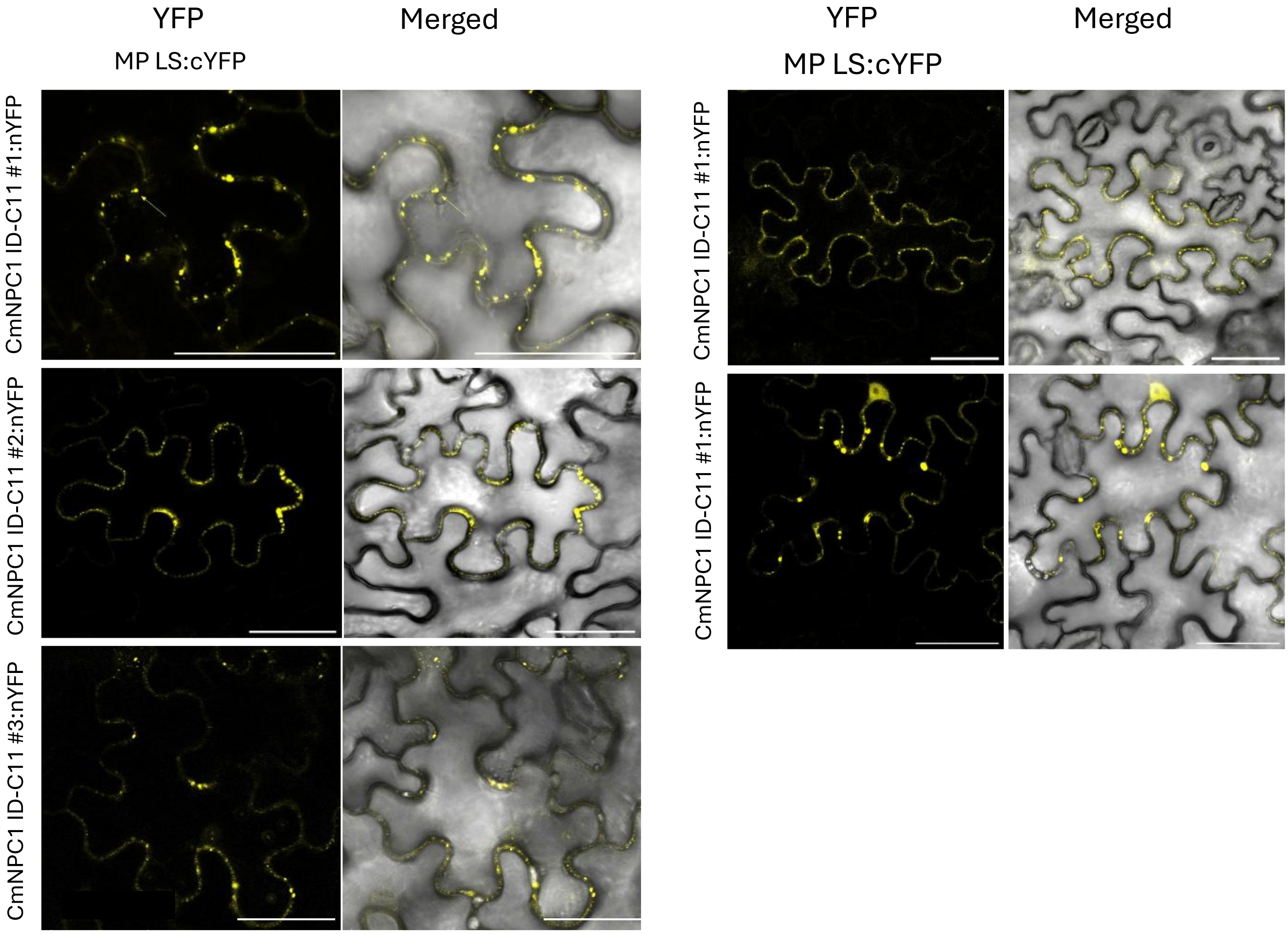

Supplementary Figure S5. BiFC assays in leaf epidermal cells showing interaction between CmNPC1 ID-C11 #1-#5:nYFP and MP LS:cYFP. "Merged": YFP and bright field channel together. Representative image from three leaves in three independent experiments. Scale bar = 50 µm.
