# Supplementary material for: The Melon Sterol Transporter Niemann-Pick C1 Protein Is a New Interactor of Cucumber mosaic virus Movement Protein
Source: Viruses. 2026 May 20;18(5):577. doi: 10.3390/v18050577 (PMC13211540; doi:10.3390/v18050577)
Supplement: Supplementary file 1 [file viruses-18-00577-s001.zip › Supplementary figure S6.pdf]

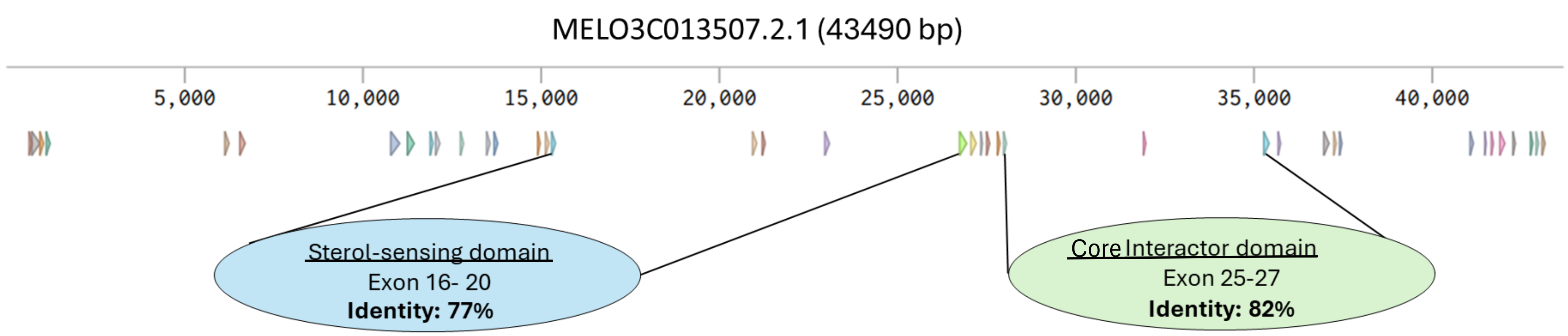


```
CmNPC1-11      GATATCCTTACAATTGCTGTAAGCTACCTCGTTATGTTTGCCTATATATCGGTGGCTTTG 60
CmNPC1-7       GATGTCATCACGATCTTGATAAGCTATCTTGTGATGTTTGCTTACATATCTCTAACTTTG 60
               *** ** * ** **     ******* ** ** ******** ** *****  *  *****

CmNPC1-11      GGAGATTCAAATATTTCGTCGTCTTTTTACCTTTCATCCAAGGTGTTGCTTGGTCTCTCA 120
CmNPC1-7       GGTGATAGACCACATTTATCTACTTTTTACGTTTCATCAAAGGTGTTACTTGGTCTCTCT 120
               ** ***  *     **  **  ******** ******* ******** ***********

CmNPC1-11      GGAGTTATACTTGTTGTGCTGTCTGTTCTAGGATCTGTAGGATTCTTCAGTGCCATCGGA 180
CmNPC1-7       GGAGTTGTACTTGTCATGTTGTCTGTTCTTGCATCAGTAGGTTTTTTCAGTGCTATTGGT 180
               ****** *******  ** ********** * *** ***** ** ******** ** **

CmNPC1-11      ATAAAATCAACACTAATAATTATGGAGGTTATTCCATTCCTGGTGTTGGCGGTAGGAGTT 240
CmNPC1-7       GTTAAATCTACTCTAATCATTATGGAAGTCATTCCTTTCCTTGTTCTGGCAGTTGGGGTG 240
                * ***** ** ***** ******** ** ***** ***** **  **** ** ** **

CmNPC1-11      GACAACATGTGTATATTAGTACACGCTGTGAAACGACAACCATATGAGTTGTCTCTAGAA 300
CmNPC1-7       GATAATATGTGTATATTGGTACATGCTGTTAAACGGCAGTCAGTAGAATTGCCTTTGGAA 300
               ** ** *********** ***** ***** ***** **  **   ** *** ** * ***

CmNPC1-11      GATCGCATAAGCAGTGCGCTGGTTGAAGTTGGTCCTTCCATAACATTAGCTAGTTTGTCA 360
CmNPC1-7       GGACGAATTAGTAATGCACTTGTAGAAGTTGGACCATCCATAACACTAGCAAGTTTATCT 360
               *  ** ** ** * *** ** ** ******** ** ********* **** ***** **

CmNPC1-11      GAGATCTTGGCTTTTGCCGTTGGAACTTTTGTTCCGATGCCAGCATGCCGTGTTTTTTCC 420
CmNPC1-7       GAGGTTCTTGCATTTGCAGTTGGAAGTTTCATTCCTATGCCAGCATGTCGTGTTTTTTCC 420
               *** *  * ** ***** ******* ***  **** *********** ************

CmNPC1-11      TTGTTTGCTGCTTTGGCAGTGTTGCTCGACTTCATTCTTCAACTGTCAGCATTTGTGGCT 480
CmNPC1-7       ATGTTTGCAGCGTTAGCCGTTCTTCTGGACTTCCTGCTCCAAGTTACAGCTTTTGTTGCT 480
                ******* ** ** ** ** **  * ** ****** * ** *** *  **** ***** ***

CmNPC1-11      CTTATAGTGTTA   492
CmNPC1-7       CTAATAGTATTT   492
               ** ***** **
```

```
CmNPC1-11      AGCTCTAGATCTAGACAGACAAACCAGCTGTGCTCCATCAGCCATTGTGATTCAAACTCC 60
CmNPC1-7       AGCTCAGAATCAAGACAGACAAACCAATTATGCTCCATCAGTCAATGTGACTCAGACTCT 60
               *****   *** **************  * *********** ** ***** *** ****

CmNPC1-11      CTGTTGAATGAGATATCAAGAGCATCATTGACACCAGAGTTGAACTACATTGCTAAACCA 120
CmNPC1-7       CTTTTAAATGAGATTGCAAAAGCTTCACTAATACCAGAGTCGAGTTTCATTGCCAAACCA 120
               ** ** ********  *** *** *** * * ******** **  * ****** ******

CmNPC1-11      GCAGCATCATGGCTCGATGATTTTCTTGTCTGGTTGTCTCCAGAGGCATTTGGTTGCTGC 180
CmNPC1-7       GCTGCTTCATGGCTTGATGATTATCTTGTGTGGATATCTCCCGAAGCATTTGGATGCTGT 180
               ** ** ******** ******* ****** *** * ***** ** ******** *****

CmNPC1-11      CGGAAATTTACAAATGGTTCTTATTGTCCTCCTGATGACCAGCCTCCTTGCTGTTTT    237
CmNPC1-7       CGGAAGTTCACAAATGGGAGTTATTGCCCTCCTGATGATCAGCCCCCTTGCTGTACT    237
               ***** ** ********   ****** *********** ***** *********  *
```

**Supplementary Figure S6.** Alignment of DNA sequences for the Sterol-Sensing Domain (SSD) and Core Interactor Domain (CID) of *CmNPC1-C7* and *CmNPC1-C11*.
