# Supplementary material for: The Melon Sterol Transporter Niemann-Pick C1 Protein Is a New Interactor of Cucumber mosaic virus Movement Protein
Source: Viruses. 2026 May 20;18(5):577. doi: 10.3390/v18050577 (PMC13211540; doi:10.3390/v18050577)
Supplement: Supplementary file 1 [file viruses-18-00577-s001.zip › Supplementary Figure S7.pdf]

A

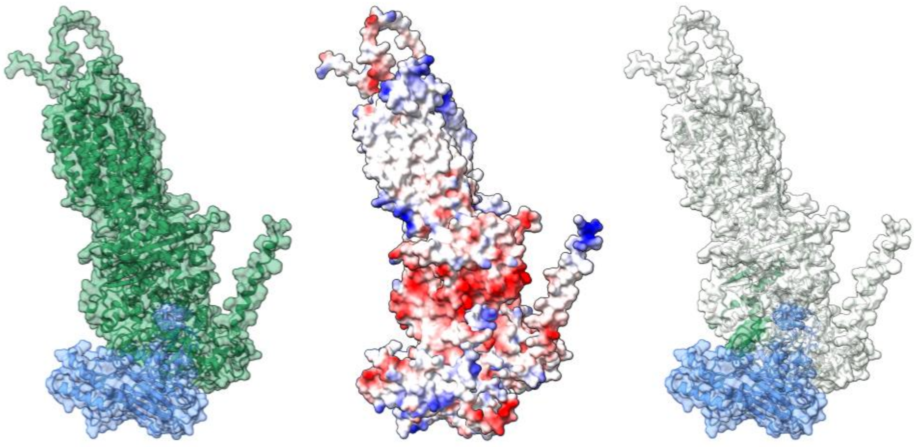

B

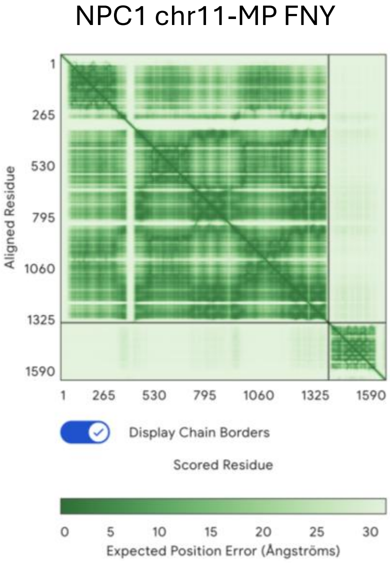

Supplementary Figure S7. Alphafold model of the interaction between CmNPC1-11 and CMV FNY MP. (A) Left: Surface and ribbon representation of the complex, with CmNPC1-C11 (green) and MP-FNY (blue). Middle: Coulombic electrostatic potential surface of the complex (red, negative; blue, positive; white, neutral). Right: Highlighted view of the interaction domain (residues 909–991) within the CmNPC1 scaffold (white). (B) PAE plots for the NPC1-MP complex. The plots indicate the expected positional error for all residue pairs, where dark green regions represent high confidence in relative positioning. The dark blocks along the diagonal correspond to the internal folding of individual chains, while the lighter off-diagonal regions represent the predicted interface and relative orientation between NPC1 and MP
