# Supplementary material for: The Melon Sterol Transporter Niemann-Pick C1 Protein Is a New Interactor of Cucumber mosaic virus Movement Protein
Source: Viruses. 2026 May 20;18(5):577. doi: 10.3390/v18050577 (PMC13211540; doi:10.3390/v18050577)
Supplement: Supplementary file 1 [file viruses-18-00577-s001.zip › Supplementary Figure S8.pdf]

CmNPC1-C11 ***CmNPC1-C7***

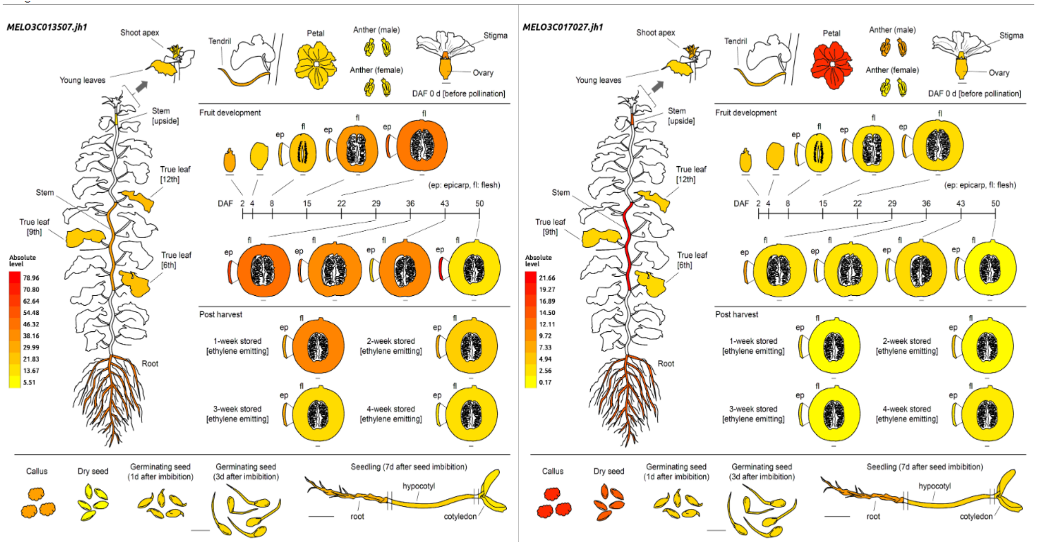

Supplementary Figure S8: . Expression atlas of both *CmNPC1* genes in different melon tissues. A darker color indicates higher expresión.
