# Supplementary material for: The Melon Sterol Transporter Niemann-Pick C1 Protein Is a New Interactor of Cucumber mosaic virus Movement Protein
Source: Viruses. 2026 May 20;18(5):577. doi: 10.3390/v18050577 (PMC13211540; doi:10.3390/v18050577)
Supplement: Supplementary file 1 [file viruses-18-00577-s001.zip › Supplementary Table S1.pdf]

Supplementary Table S1. Primers used in this study. In bold: BamHI (5') and EcoRI (3') restriction sites.

Primer name	Sequence (from 5' to 3')	Experiment
MP-EcoRI-F	CATGGAGGCC**GAATTC**ATGGCTTTCCAAGGTACCAGTAGG	Y2H screening
MP-BamHI-R	GCAGGTCGAC**GGATCC**AAGACCGTTAACCACCTGCGGTCT	Y2H screening
CMV-2F	TAATACGACTCACTATAGGGC	Y2H screening
CMV-2R	AGATGGTGCACGATGCACAG	Y2H screening
NPC1-YN-F (attB1)	GGGGACAAGTTTGTACAAAAAAGCAGGCTGCATTTTGTTGTGAAGGATTACAATTATAG	BiFC
NPC1-YN-R (attB4)	GGGGACAACTTTGTATAGAAAAGTTGGGTGTTTGTAACTTTTAGTGTGTGTGAAAA	BiFC
L-Ascorbate-YN-F	GGGGACAACTTTTCTATACAAAGTTGTGATGAGGGAATACAGAGTACTCTGTTCT	BiFC
L-Ascorbate-YN-R	GGGGACAACTTTGTATAGAAAAGTTGGGTGCTTGGAAGCAGCAGTAGTACATAA	BiFC
NPC1 attb1	GGGGACAAGTTTGTACAAAAAAGCAGGCTGCTATTTTGTTGTGAAGGATTACAATTATAGC	Co-IP
NPC1 attb2	ACCACTTTGTACAAGAAAGCTGGGTATTGTTTTAGGGTTTTTCACATAAACCTAG	Co-IP
MP attb1	GGGGACAAGTTTGTACAAAAAAGCAGGCTCGATGGCTTTCCAAGGTACC	Co-IP
MP attb2	GGGGACCACTTTGTACAAGAAAGCTGGGTAAAGACCGTTAACCACCTG	Co-IP
NPC1-7ID attB4r F	GGGGACAACTTTTCTATACAAAGTTGGCATCTCAGAATAGGTCCACCGGTAT	BiFC
NPC1-7 ID attB2 R	GGGGACCACTTTGTACAAGAAAGCTGGGTAGTGAAGAAAGCACGTTGTACAATC	BiFC
