# Supplementary figures and images for: The Melon Sterol Transporter Niemann-Pick C1 Protein Is a New Interactor of Cucumber mosaic virus Movement Protein
Source: Viruses. 2026 May 20;18(5):577. doi: 10.3390/v18050577 (PMC13211540; doi:10.3390/v18050577)

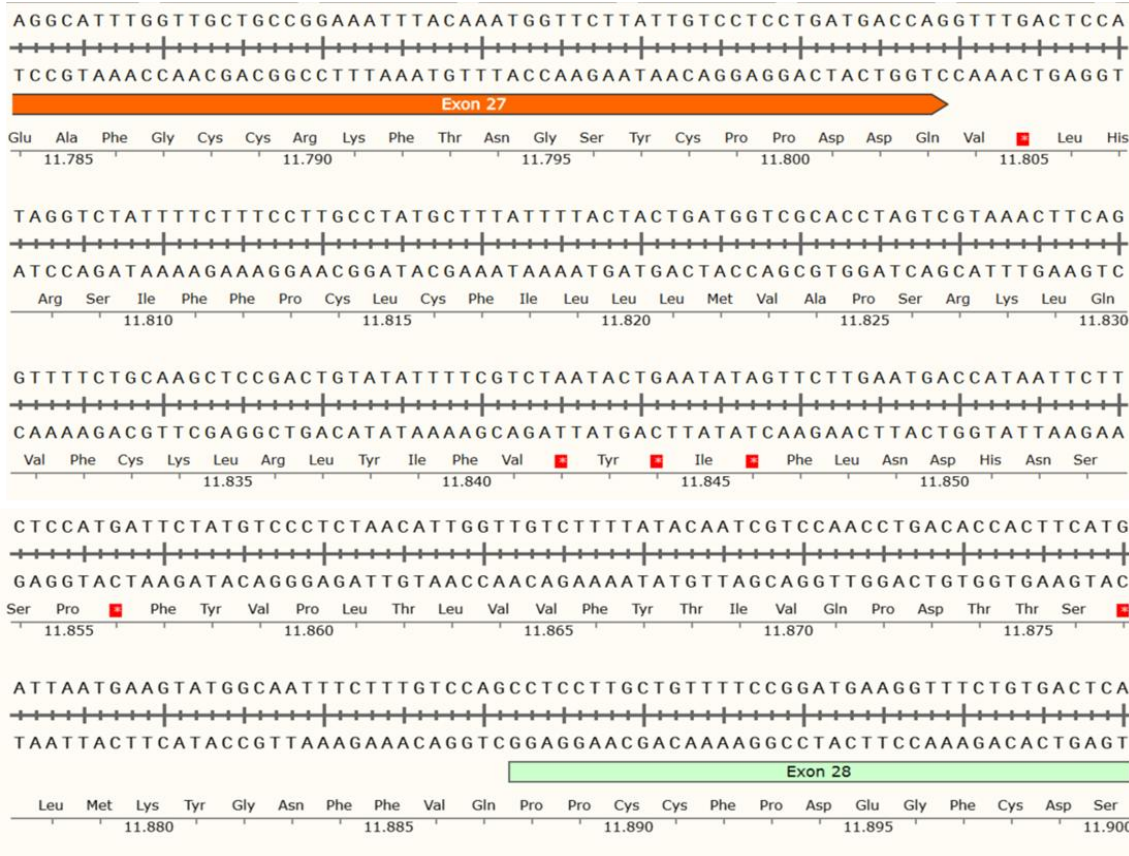

**Supplementary Figure S4.** In frame stop codons present in intron 27-28 of CmNPC1-C11.

Supplement: Supplementary file 1 [file viruses-18-00577-s001.zip › Supplementary Figure S4.pdf]
